# Moving Object Detection in Heterogeneous Conditions in Embedded Systems

**DOI:** 10.3390/s17071546

**Published:** 2017-07-01

**Authors:** Alessandro Garbo, Stefano Quer

**Affiliations:** Dipartimento di Automatica ed Informatica, Politecnico di Torino, 10129 Torino, Italy; alessandro.garbo@polito.it

**Keywords:** motion estimation, human detection, tracking, embedded systems, automatic surveillance

## Abstract

This paper presents a system for moving object exposure, focusing on pedestrian detection, in external, unfriendly, and heterogeneous environments. The system manipulates and accurately merges information coming from subsequent video frames, making small computational efforts in each single frame. Its main characterizing feature is to combine several well-known movement detection and tracking techniques, and to orchestrate them in a smart way to obtain good results in diversified scenarios. It uses dynamically adjusted thresholds to characterize different regions of interest, and it also adopts techniques to efficiently track movements, and detect and correct false positives. Accuracy and reliability mainly depend on the overall receipt, i.e., on how the software system is designed and implemented, on how the different algorithmic phases communicate information and collaborate with each other, and on how concurrency is organized. The application is specifically designed to work with inexpensive hardware devices, such as off-the-shelf video cameras and small embedded computational units, eventually forming an intelligent urban grid. As a matter of fact, the major contribution of the paper is the presentation of a tool for real-time applications in embedded devices with finite computational (time and memory) resources. We run experimental results on several video sequences (both home-made and publicly available), showing the robustness and accuracy of the overall detection strategy. Comparisons with state-of-the-art strategies show that our application has similar tracking accuracy but much higher frame-per-second rates.

## 1. Introduction

The number of surveillance cameras in urban areas has been increasing at a rate which results in massive amounts of video to be analyzed. As a consequence, in the last decade, many approaches [1,2,3,4,5,6,7,8,9,10,11] have been proposed for moving object detection and tracking from videos. Researchers concentrate on traffic monitoring and security, visual surveillance, and sensors networks able to analyze complex area and flow movements.

Detection of moving objects in videos is based on the realistic assumption that in almost all frames those objects are perceivable as different from the background and from recent previous frames. When models of target objects are known a priori, model-based applications can be used for improving detection. On the contrary, when precise geometrical models are not possible (for instance, for non-rigid objects) or, simply, object models are not exhaustively known in advance, model-based detection cannot be applied. When the analysis concentrates on moving people, it involves very complex situations, as tracking must follow heterogeneous trajectories over time. In those cases, to perform tracking it is usually necessary to find feature-based correspondences of the same physical objects in different frames. As a consequence, robust human tracking is highly dependent on reliable detection in each frame, and most of the existing approaches are unsuitable for detecting targets with large variance in appearance. Therefore, robust human detection remains a challenge.

In this work, we focus on a system which detects and tracks different and possibly unknown objects; it restricts its attention to pedestrians, tracking and finally counting them. We will describe all main steps from the video analysis to the pedestrian tracking phase. Even if those steps do share several aspects and state-of-the-art methodologies with previous approaches, our system orchestrates them in a new way presenting an innovative recipe to reach the final goal.

Our application is divided into three separated modules including several concurrent threads. To maximize concurrency, threads are organized as a multi-layer pipeline, where each layer manipulates information coming from the previous layers and it delivers more compact data to the next layers.

The first module is in charge of partitioning the problem into sub-problems and dividing the overall workload among all other threads. Partitioning is performed adopting an eager divide-and-conquer approach. In more detail, given a video frame *V*, each frame *F* is partitioned into a grid of cells and different sub-sets of those cells are fed to the different modules/threads. Great care is taken to partition the grid and to select sub-sets in an efficient way, such that subsequent computation tasks are more efficient and accurate.

The second module statically analyzes each cell to perform background subtraction, followed by static and a dynamic luminosity analysis, and finally movement detection. The target of this phase is to allow the third module to focus only on promising areas of the videos where some movement has already been detected, so saving time and increasing accuracy. Those phases are mainly implemented using well-known algorithms optimized such that they efficiently manipulate our grid cells. Moreover, we use histograms to statistically represent and analyze texture pixels, and dynamically adjusted thresholds to avoid false detections.

The third module uses information coming from the previous one to create reliable bounding boxes around relevant points. Points with similar dynamic characteristics are then grouped into entities which we call “swarms”. Swarms are then tracked, updated, merged, and eventually ruled out depending on the set of points belonging to their support. In an initial transitory phase, the system also develops a model to subsequently correct swarm tracking. This model, called the “hole model”, allows the system to recover correct information whenever tracked points behave erratically. This may be due to background “anchors” attracting moving points into frozen positions. Substantially, the model corrects errors with sophisticated blob analysis and restoring techniques.

Great care is also taken to leverage all computational phases, to make threads to efficiently cooperate to reach the final goal, and to keep the computational effort low during all phases. To sum up, the system presents the following main characterizing features and contributions:

1. From the object tracking point of view we substantially have a three-phase approach. During the first phase, we partition the problem. During the second, we intercept static frame-by-frame movements using histograms and dynamically adjusted thresholds. During the third phase, we detect anchor points on the background, and we use a hole model to check for and correct errors made during the final swarm tracking phase.
2. Our multi-layer and multi-thread pipelined application is suitable for embedded systems, with real-time requirements and finite computational resources, such as computational power, memory, and energy availability.
3. Keeping in mind the previous point, the application shows an average accuracy comparable with other state-of-the-art approaches, but higher frame-per-second rates. Moreover, it also shows very limited memory (of all types) requirements, as it is usually demanded for embedded applications.
4. The system is conceived to be auto-adaptive, i.e., to work with the best possible performances on all possible scenarios without any sort of initial or on-line manual set-up. Results show that accuracy results are aligned with other state-of-the-art approaches, but they are more stable in all corner-case situations.
5. Our application upgrades a system composed of a single video camera and a single central computing workstation to an intelligent network grid. Each node of the network is equipped with a single off-the-shelf fixed camera, and an inexpensive embedded system, autonomously performing many computations locally on the node. In this way only a reduced amount of tracking information is transferred to the control room, then preventing heavy computations (on data coming from several cameras) for the central server. As a consequence, the central host may concentrate on pedestrian counting, flow analysis [12] or high-level grid evaluations which can be carried out based only on the fused and consolidated data coming from the network nodes.
6. The system is able to operate in different urban scenarios with no training or previously acquired models: (1) video cameras are placed at a relative high height for security, safety, and economic reasons, and the perspective may produce relevant object distortions; and (2) infrastructures, large shapes, and moving objects may cause occlusions and variable reflection conditions.

As a final remark, notice that the work has been part of an industrial project (Industrial contract 254/2015, with Telecom Italia Joint Open Lab, entitled “Pedestrian Tracking”) whose main target was to evaluate pedestrian flows (i.e., count the number of pedestrians crossing a specific street, bridge or square) in several historical area of the city of Venice, in Italy, where the touristic crowd can overwhelm tourist infrastructure. Current city plans (as publicly described on the news and on local newspapers) involve a period in which crowd flows will be examined and subsequently controlled, using several techniques like the one we will describe in this paper.

### Road-Map

The paper is organized as follows. Section 2 describes some related recent works. Section 3 illustrates our strategy from a high-level point of view. Section 4 and Section 5 introduce the two core parts, namely the static movement detector and the dynamic tracking module, reporting all main implementation details to make the work reproducible. Section 6 reports our experimental results. Finally, Section 7 draws conclusions.

## 2. Related Works and Comparisons

Pedestrian detection is a canonical instance of object detection. Because of its direct application to car safety, surveillance, and robotics, it has attracted much attention in the last years. As such, it has served as a playground to explore different ideas for object detection. Among the seminal works, it should be remembered that the detector of Viola and Jones [13] has been applied to the task of pedestrian detection. A broader attention on the topic was brought by Dollar et al. [14] who introduced the Caltech Pedestrian Dataset which was two orders of magnitude larger than existing datasets. The established methodology for evaluating pedestrian detectors also changed from per-window (FPPW) to per-image (FPPI), once the flaws of the per-window evaluation were identified. Many works have been published from that publication on, reflecting a renewed interest in the problem. Nowadays, it is possible to discern three families of pedestrian detection methods: Deformable Part Model (DPM) variants, deep networks, and decision forests.

Dalal and Triggs introduced the landmark Histogram of Oriented Gradients HOG [15] detector, which later served in 2008 as a building block for the now classic DPM. The DPM [16,17,18] combines rigid root filters and deformable part filters for detection, and it performs well for high resolution objects. Anwer et al. [19] evaluated the opponent color space as an alternative to RGB space for human detection. As the authors obtained better detection performance, they suggested it could be worthwhile computing co-occurrence matrices, self-similarity features, etc., also on top of opponent space as usually done with HOG. Xu et al. [20] used the DPM for representing objects, and focused on performing an incremental domain adaptation of object detectors. The main benefit was to improve existing source-oriented detectors as soon as a small amount of labeled target-domain training data is available, and keep improving as more of such data arrives in a continuous fashion.

Decision forests [21,22,23] seem particularly suited for pedestrian detection, reaching top performances on several benchmark sets. It is unclear however what gives them an edge. For example, to obtain a successfully detector González et al. [24] provide an extensive evaluation along three orthogonal axes: (1) the integration of multiple feature cues; (2) the fusion of multiple image modalities; and (3) the use of multiple views of the pedestrian. Their analysis revealed that, although these aspects are important, the fusion of visible spectrum and depth information allows for boosting the accuracy significantly by a large margin.

In recent years, deep networks [25,26] have been applied to pedestrian detection, achieving promising results and fast progress in detection quality. Instead of using handcrafted features, they can automatically learn features in an unsupervised or supervised fashion. Redmon et al. [27] proposed You Only Look Once (YOLO), a system based on a single convolutional network which provides a threshold for the resulting detections by the model’s confidence. YOLO is extremely fast as it is able to process images in real-time at 45 frames per second (155 flames for Fast YOLO, i.e., a smaller version of the network). Liu et al. [28] used a single deep neural network to detect objects without re-sampling pixels or features for bounding boxes. The authors proved that their method has competitive accuracy with methods adopting additional object proposal steps, and it is much faster, while providing a unified framework for both training and inference.

Within this general framework, the works closest to ours are the as follows. Cucchiara et al. [4] presented Sakbot, a system for moving object detection and tracking. The system is endowed with robust techniques, such as the statistical and knowledge-based background update, the use of Hue Saturation Value (HSV) color information for shadow suppression, and symbolic reasoning for tracking. Leone et al. [5], in order to detect potential shadow points, evaluated, for all moving pixels, the compatibility of photometric properties with shadow characteristics. Shadow detection is improved by evaluating the similarity between little textured patches. The algorithm is designed to be unaffected by the scene, the background type, and by the light conditions. Notice that two previous works methods work at a pixel level whereas as we used low-cost video cameras and embedded systems with bounded computation resources, we perform background manipulation by splitting the scene into sub-regions of fixed size, resorting to the divide-and-conquer paradigm. Mason et al. [29], and Varcheie et al. [30], to model the background and detect motion, used color histograms, texture information, and successive division of candidate rectangular image regions for applying Gaussian mixture background modeling. One of the problems with these approaches is the necessity for mandatory capture of a static background at the beginning of the process. Moreover, the authors need to optimize the color histogram generation, which original uses $2^{24}$ classes for a standard RGB pixel representation. On the contrary, we do not use an initial learning phase, and, again for efficiency reasons, we use much smaller and memory inexpensive histograms.

## 3. Designing the Application

**Figure 1:**
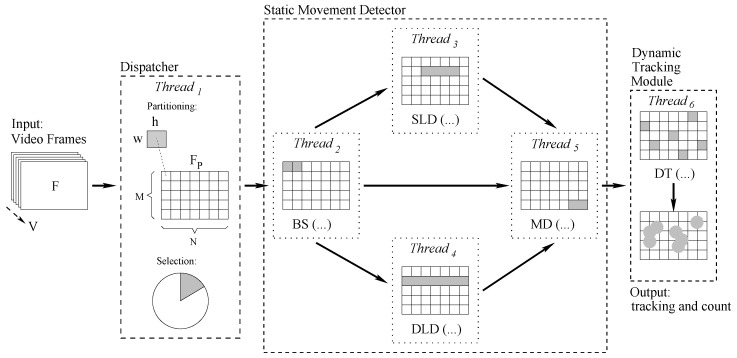
Overall information flow of our algorithm (from left to right): (1) The input video frame *F*; (2) The dispatcher, i.e., a divide-and-conquer partitioning module; (3) The static movement detector (running in parallel sub-modules). BS: background subtraction; SLD: static luminosity detector; DLD dynamic luminosity detector; MD: movement detector. (4) The dynamic tracking (DT) module.

As cheap of-the-shelf embedded systems are unable to manage the entire object tracking chain for every video frame at run-times, our application is divided into several modules running as separate concurrent threads. To maximize concurrency, threads are organized as a multi-layer pipeline, where each layer manipulates information coming from previous layers and it delivers more compact data to next layers. To further speed-up the process for embedded architectures, and to deal with entire (640×480) frames, the application also heavily adopts partitioning, following a eager divide-and-conquer approach. A pictorial representation of the overall logic flow is reported in Figure 1. All main modules (the Dispatcher, the Static Movement Detector, and the Dynamic Tracking Module) include one or more working threads, and are described in the following subsections.

### 3.1. The Dispatcher

Given a video frame *V*, a first module (running a single working thread *Thread${}_{1}$*) applies the following divide-and-conquer scheme.

First of all, it partitions each frame F∈V into a cell grid $F_{P}$ of (N×M) cells of (w×h) pixels. From a theoretical point of view, the smaller the cells, the more accurate our algorithm tends to be. At the same time, small cells require more expensive computations. From a practical point of view, it is difficult to go below or above a certain cell size, because cells lose their representativity value depicting too refined or too coarse-grained information. The cell size also depends on the perspective used to analyze the scene, as detecting movements of small objects requires smaller cells than detecting movements on large objects. In the experimental result section, we will show results with the best trade-off we found for our purpose, that is, for (640×480) pixel images, N=M=16, corresponding to cells of (w=40)×(h=30) pixels.

Secondly, it feeds all subsequent modules (and working threads *Thread${}_{2}$*-*Thread${}_{6}$*) with a non-overlapping subset, of variable size, of the cells chosen from the grid $F_{P}$. As those modules do not form an ideal pipeline, and have mutual relationships, those subsets are selected adopting the following strategy. First of all, all threads must iterate on all cells of the video frame, but they are run once and only once on each video frame. If a thread (such as background substration (BS), static luminosity detector (SLD), dynamic luminosity detector (DLD), or movement detector (MD)) manipulates a subset of $F_{P}$ of SIZE cells at each step, it will require (N×M)/SIZE frames to inspect the entire context. Then, given the dimension SIZE of the selected sub-set of $F_{P}$ to be fed to each thread, we define the “refreshing rate” as (N×M)/SIZE, i.e., the frequency used to update each cell. For example, with SIZE=4, the refresh rate is 64, i.e., cell data in every cell is is manipulated (refreshed) every 64 frames. With a frame rate of 20 frames per second this is equivalent to 3.2 s. Similarly, with SIZE=16, the refresh rate is 16 frames, and we need 0.8 s to update each cell. Moreover, threads have to manipulate information in a specific order, i.e., background subtraction has to run before the static and dynamic luminosity procedure, etc. As we choose different subsets of cells for each thread function, usually each procedure manipulates cells managed by another function sometimes before, i.e., on a previous video frame. We associate to each data (depending on its nature) a “soundness time window”, i.e., a time window in which the data is guaranteed to be meaningful. We are always guaranteed to manipulate data within its soundness time window. Functions do use synchronization primitives to manage critical sections, but our selection strategy strives to minimize all synchronization waiting times. Secondly, to choose a proper refreshing rate, we identify events requiring small response times (such as the ones involving shadow modification due to clouds, pedestrian standing times, etc.), and changes requiring long response times (such as shadow modifications due to hearth rotation, etc.). At the same time, there are algorithmic phases more expensive and other less expensive from the computational point of view. In the experimental section, we will set different values for the variable SIZE for each thread, depending on those parameters. Moreover, cell management may be optimized adopting a few further strategies. For example. using the locality principle, it is possible to select cells where movements are less common less frequently than cells where movements are more common. Furthermore, we can use statistical analysis to follow pedestrian lanes whenever possible, e.g., to iterate on the frame grid following a column-major order instead of a row-major order.

### 3.2. The Static Movement Detector

Once the Dispatcher module has partitioned the current frame *F* into $F_{P}$, and distributed the work load to all next level working threads, the Static Movement Detector module performs all major tasks to detect and track movements on a frame-by-frame basis. This means that all threads belonging to this module essentially work without memory, i.e., they analyze single video frames without considering any correlation among them. To be more precise, the module encompasses the following operations.

The background subtraction (BS) thread finds accurate and stable RGB and HSV models to be used by other threads to compute histograms. In our tool, we use a Gaussian mixture-based background/foreground segmentation algorithm [31,32], modeling each background pixel by a mixture of Gaussian distributions. This procedure is described in Section 4.1.

The static luminosity detector (SLD) thread follows Varcheie et al. [30] to assign each grid cell to a class storing cells with isomorphic luminosity levels. We use gray histograms to discriminate among luminosity classes, and we define classes with variable and adjustable sizes. This phase improve the accuracy of the subsequent movement detection phase which can be heavily influenced by the luminosity level within the cell. This function is analyzed in Section 4.2.

The dynamic luminosity detector (DLD) thread detects notable rapid luminosity changes on the grid. Following Boreczky et al. [33], every time the conditions in a cell are changing rapidly we mark the cell as in transient state and manipulate it separately by standard cells. This step reduces false detections, i.e., it helps to discern between simple luminosity changes and real movements. This phase is analyzed in Section 4.3.

The movement detector (MD) threads collects outputs coming from previous threads, such as histograms, thresholds, etc., to detect notable movements in a cell. Unlike many other algorithms, this thread works at a cell level instead of concentrating on single pixels. It essentially set a movement flag on each cell where movements have been detected, such that the dynamic tracking module will be able to focus on those cells only. This thread is discussed in Section 4.4.

### 3.3. The Dynamic Tracking Module

All threads belonging to the static movement detector essentially work without memory, i.e., they analyze cells in each single video frame without correlating data coming from different frames in any way. On the contrary, the dynamic tracking module grasps the correlation between subsequent video frames. First of all, it concentrates its attention only on interesting cells, i.e., the ones detected by the static movement detector module as including “static” movements. Then, it performs object tracking following [34,35,36,37] and using information coming from the MD module.

Points with similar dynamic characteristics are grouped into entities which we call swarms. Swarms are then tracked, updated, merged, and eventually ruled-out depending on the set of points belonging to their support. In an initial transitory phase, the system also develops a model to subsequently correct swarm tracking. This model, called hole model, allows the system to recover correct information whenever tracked points behave erratically. This may be due to background “anchors” attracting moving points into frozen positions. Substantially, the model corrects errors with sophisticated blob analysis and restoring techniques.

The dynamic tracking system finally represents movement information and send it to the host computer, which in turn is free to deal with the entire sensor grid to perform pedestrian counting, flow analysis and compute statistical measures. This thread is discussed in Section 5.

## 4. Static (without Memory) Movement Detector

**Figure 2:**
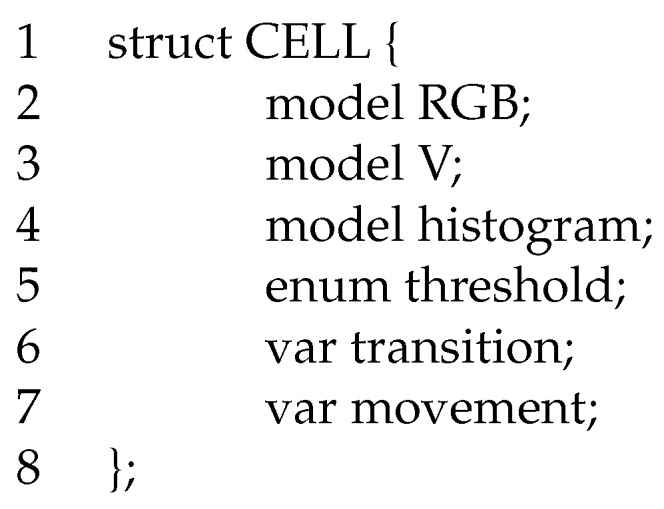
Cell data structure.

To allow some sort of data sharing among the different steps of the algorithm, we associate to each cell an abstract data type (ADT) structure with the data fields reported in Figure 2. We will consider ADT as globally visible or eventually embedded in a class object, with proper synchronization access primitives on all setter and getter methods. All fields will be described in the following sub-sections, but essentially they store the coming data. The first two fields of Figure 2, RGB and a V (lines 2–3), are internal data structures, representing the RGB channel and the V channel of the HSV background model (see Section 4.1).

The easiest way to represent texture information in a histogram is to encode color or gray information [29,30]. For this reason, we will use two histograms, the first one represented in Figure 2, and the second one introduced in Section 4.2. Our histograms are arrays of 255 integer values, representing gray tones differences or pure gray tones. Each bin is strictly related to the data represented by the texture grid, as it indicates the number of pixels in the cell with a specific gray value. As in our implementation, each cell of the partitioned frame $F_{P}$ has a size equal to (40×30) pixels, the sum of all values stored in those bins will be 1200 (see Section 4.2 and Section 4.3).

The threshold data field (line 5) is an enumerated data type (enum) storing a set of possible threshold luminosity values (such as, black, dark, dim, sunrise, bright, sunny, heat) that we will use throughout the paper. Fields on lines 6 and 7 are flags (or back-counters, i.e., timers) used to indicate that a transition, in terms of luminosity or movement, respectively, has been detected in the cell (see Section 4.4).

### 4.1. Building a Background Model: Function BS

**Figure 3:**
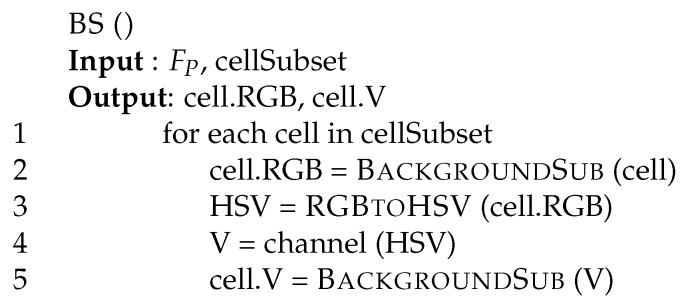
Background subtraction: function BS.

As in many other approaches, we need an accurate and stable background to catch foreground movements. Background subtraction (BS) is a common and widely used technique for generating a foreground mask performing a subtraction between the current frame and a background model. In contrast to many other approaches, we rely on a standard implementation of the background subtraction phase processing as reported in Figure 3.

The procedure receives as input the video frame $F_{P}$, and the sub-set of cells it has to manipulate (cellSubset). For each cell in the selected set of cells cellSubset (line 1), the thread calls procedure BackgroundSub. While coding, we are mainly interested to create the background object and not the foreground mask (namely, a binary image containing the pixels belonging to moving objects in the scene) generated by the method. In our tool, we use a Gaussian mixture-based background/foreground segmentation algorithm [31,32], modeling each background pixel by a mixture of K (from 3 to 5) Gaussian distributions. As we want to keep the system quite reactive, we set the history length to 10 frames. For example, with SIZE=4, a refresh rate of 64, and a frame rate of 20 frame per second, each cell cell is refreshed every (64×20)=3.2 s, and a history equal to 10 implies storing all past frames up to 32 s. Background objects are assigned to the RGB field of the CELL data structure in Figure 2 (line 2).

From line 3 on, following Cucchiara et al. [4] and Leone et al. [5], we implement a shadow detector performing background subtraction on the V channel of the HSV coding [38,39,40]. Usually, for efficiency reasons, the shadow suppressor is activated only on cells where the luminosity level allows shadows to be present, i.e., not on too dark or too clear cells. This means that for the V model, we automatically update only the background model, whereas the foreground is updated only on request (lines 3–5). Function BS finally returns the RGB and the V background models for the cells (namely, fields cell. RGB and cell. V of Figure 2).

### 4.2. Dealing with Static Luminosity Information: Function SLD

Varcheie et al. [30] track movements by comparing color histograms using a threshold-based strategy. In unfriendly open areas, where luminosity conditions may vary a lot during the day, thresholds have to be selected in a very dynamic fashion. As a consequence, we manipulate in different ways cells with different luminosity levels, and we use gray histograms to divide cells into classes with similar luminosity levels (gray tones of all pixels within a cell are stored in the histogram model, introduced in Section 4).

**Figure 4:**
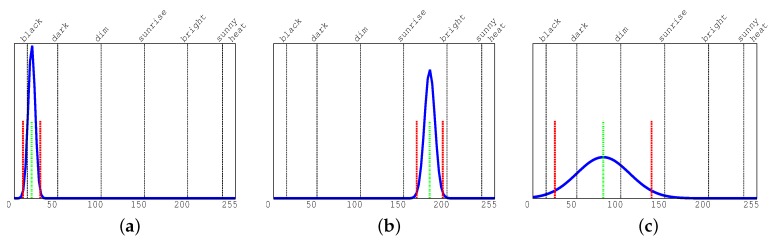
Gray histograms for: (**a**) a uniform dark cell, (**b**) a uniform bright cell, (**c**) a non-uniform dark cell. Vertical lines represent average (green) and variance (red) on all bins.

Figure 4, shows a few examples on how we use gray histograms to discriminate among isomorphic luminosity classes. On all histograms, the three smaller vertical lines represent the cell average value and the variance on all bins (the green and red lines, respectively, on color printing). All other (higher) vertical lines identify our classes with isomorphic luminosity. We define those classes as black, dark, dim, sunrise, bright, sunny, and heat. To have the highest possible representativity, they do not have the same size. For example, the first and the last classes are smaller than the other ones because they represent extreme working conditions for the video camera (with very low or high luminosity). At the same time, the central classes (named dim, sunrise and bright) are the most representative for standard conditions in our videos. Function threshold (line 5) selects the class to which a cell belongs to, comparing with our thresholds the value of the variance on the right of the average value. Among those classes, we will manipulate uniform and non-uniform cells in a different way. Notice that in our context, the term uniform is used to indicate small luminosity variance within the same cell.

**Figure 5:**
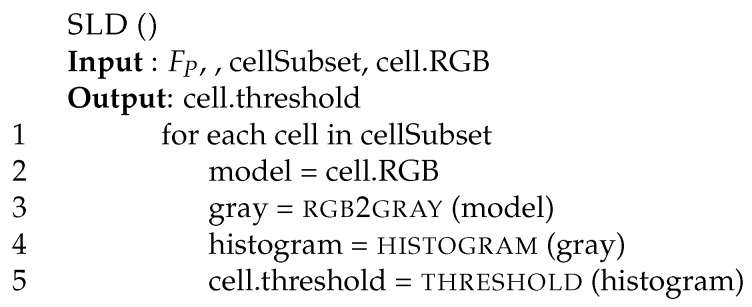
Static luminosity: function SLD.

Figure 5 reports the pseudo-code dividing cells into classes based on their static luminosity. The procedure receives as input the video frame $F_{P}$, the sub-set of cells to manipulate (cellSubset), and the cell RGB model (cell.RGB) computed by function BS. It produces for each cell a gray histogram and a luminosity threshold level as follows. Function SLD converts the RGB representation of the background into a gray scale (line 4). The gray value is an integer value (in the range [0,255]) representing the pixel gray level. Then, it builds a gray histogram for each cell (stored in the local variable histogram) where each bin represents the number of pixels (out of 30×40) with the same gray tone. At the same time, as the histogram may be represented as a discrete Gaussian curve, it computes its average and variance values. Those values are then used to divide cells into classes of isomorphic luminosity (line 6).

### 4.3. Dealing with Dynamic Luminosity Information: Function DLD

In this section, we describe how we take care of rapid luminosity modifications, such as the ones caused by rapid weather changes.

**Figure 6:**
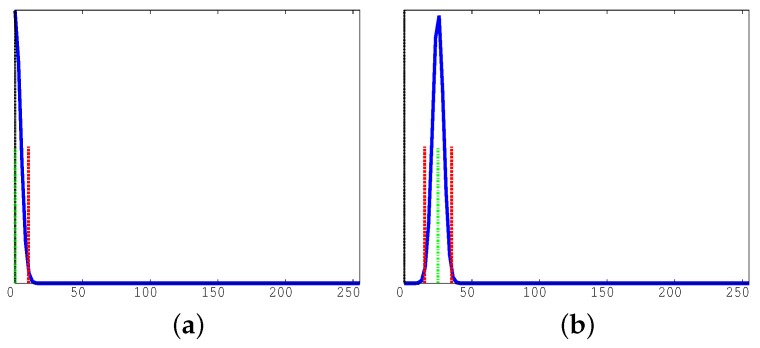
Difference histograms for: (**a**) a cell with no notable background alteration, (**b**) a cell with a rapid luminosity transition. In the second case there are no Gaussian points in the origin. Vertical lines represent average (green) and variance (red) on all bins.

As described by Boreczk et al. [33], the histogram of the difference between the current RGB pixel value and the model computed during the background subtraction phase can be used by module DLD to detect dynamic luminosity changes. Unfortunately, when there are rapid and uniform changes in luminosity those changes can generate false movement detections. To avoid false detection, Figure 6 illustrates how the histogram behaves when there is a rapid luminosity transition without any foreground movements. Figure 6a shows cases in which there are no notable alterations of the background, whereas Figure 6b represents a situation in which there is a rapid luminosity change. The average value is larger for more abrupt changes. Notice that when the transient DLD histogram response is finished and we are back to a steady state, the histogram configuration moves back to the one of Figure 6a. During the transitional period the module DLD essentially issues a warning on the cell or set a timer to a specific value indicating that movements have to be evaluated after a few frames with larger thresholds.

**Figure 7:**
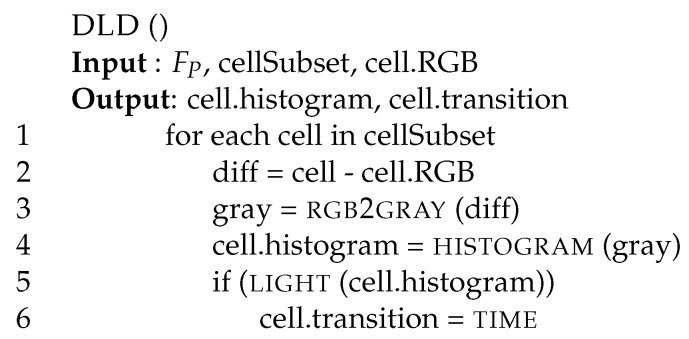
Dynamic luminosity: function DLD.

Figure 7 reports the pseudo-code of our implementation of the DLD module. The procedure receives as input the video frame, the sub-set of cells it has to manipulate, and the RGB model for each cell (cell.RGB). For each cell, it computes the difference between the RBG representation of each pixel of the current video frame and the representation of the background model (line 2). After that, it converts the previous difference (diff) into gray tones (gray, line 3) using function rgb2gray. The gray tones are integer values included in the [0,255] range. After that, it computes the histogram of such differences (line 4) using function histogram [41]. Finally, if this histogram stores any value larger than 0 in the first 5 bins (function light, line 5), the procedure sets the transition field of that cell (cell.transition) equal to the integer value time (line 6). This indicates that the cell is in a transient luminosity condition.

### 4.4. Detecting Movements on the Grid: Function MD

While all previous modules work to maintain all data updated, i.e., they work to create a proper “soundness window” for all data, the MD module collects all previously computed information to intercept movements.

**Figure 8:**
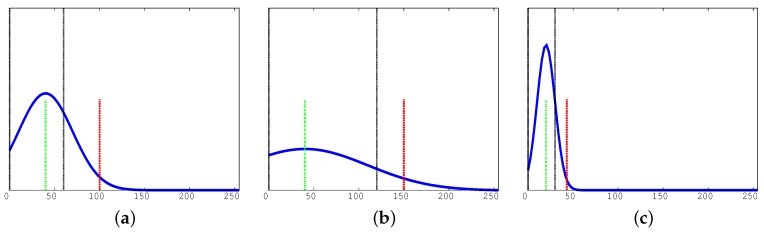
Difference histograms for cells with a detected movements: (**a**) a dark cell, (**b**) a bright cell, (**c**) a hot (white saturated, i.e., dazzled) cell. Vertical lines represent average (green), variance (red), and the cell threshold (higher black line). All Gaussian curves have points in the origin.

Unlike many other algorithms, we do not work at the pixel level, instead we focus on the cells of our grid. From the one hand, we perform a more coarse evaluation than the one available concentrating on pixels. On the other, our process is much more stable than the pixel-based one when applied in extreme conditions, such as the ones in which dark building shadows occupy part of the scene. Moreover, we can be much faster in performing all necessary computations on cells than on single pixels, and the data computed are more manageable by the higher levels of the system. Figure 8 shows how movements can be detected in different luminosity conditions. When there is no movement on a cell, the cell content is quite similar to that collected during the background subtraction. As a consequence, the histogram of the differences has an average value quite close to zero and a very small variance (as described in Figure 6a). On the contrary, when there is a movement in a cell, the histogram of the differences tends to have much larger average and variance values.

**Figure 9:**
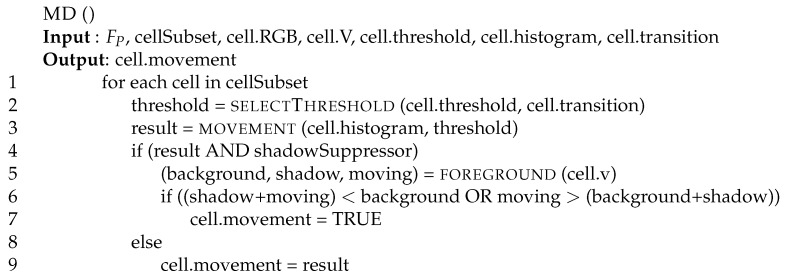
Checking for movement: function movement detector (MD).

Figure 9 reports the pseudo-code used to detect movements. The procedure receives as input the video frame, the cell sub-set it has to manage, and several cell data fields computed (and constantly updated), by all other modules. It sets the field movement of the analyzed cells to enable the tracking performed by the dynamic tracking module (see Section 5). First of all, the procedure dynamically selects a threshold (line 2) based on the class identifier (Section 4.2) and the cell transition field. We use smaller thresholds for cells with low luminosity levels and larger thresholds for cells with high luminosity levels. As a consequence, we set the current threshold equal to the cell threshold when there is no warning in the transition field, whereas the threshold is increased when there is a warning to cope with abrupt luminosity transitions. We would like to point out that the threshold value does not change linearly, as its increments are larger in intermediate conditions (dim, sunrise, and bright), and smaller in extreme conditions (such as black, dark, sunny, and heat). This threshold value is then used on line 3 to set the variable result to true if the variance is higher than the threshold. At this point we have ruled-out all noise derived from the luminosity level, but we still have to rule out false movements coming from shadows detection. If there is a possible movement on a cell allowing shadow problems (i.e., in dim, sunrise, and bright conditions), and we want to suppress shadow problems (line 4), we analyze the field HSV of the cell (cell.v) by collecting (line 5) the number of pixels in the background, in the shadow, and moving (variables background, shadow, and moving, respectively). Then, we use those values to set the field cell.movement, which is true when the background includes much more points (in this case we trust the static analysis), or when the moving pixels include more points than the background or shadow sets. Actually, cell.movement is a down-counter, as the cell where movement is detected has to remain active for a pre-defined number of frames. Then, its value is set to the pre-defined constant when a movement is detected, it is decremented on each video frame, and it considered to be true until it reaches zero.

## 5. Dynamic (with Memory) Tracking

As introduced in Section 3 our dynamic tracking module executes one more concurrent thread. This thread uses standard corner detection and optical flow algorithms, but it re-designs and optimizes them for embedded systems, focusing only on those cells where the static tracking phase of Section 4 detects some movement (i.e., cells for which the field cell.movement is true).

To track pedestrians on multiple time frames we have to create reliable bounding boxes based on relevant points. Statistically speaking, due to perspective changes, many points last only a few frames before disappearing or becoming unstable. Due to this dynamic object behavior, the system creates groups (or swarms) of points on the Region Of Interest (ROI) image computed on those cells on which some movement has been detected.

**Figure 10:**
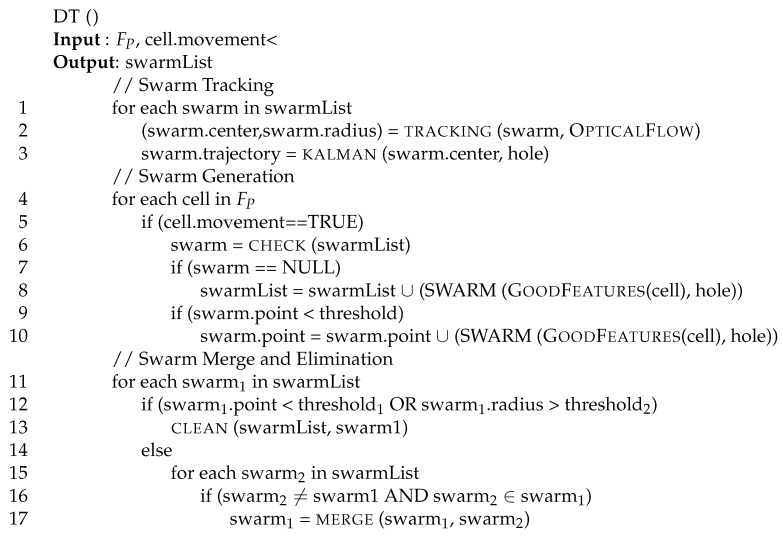
Dynamic tracking (DT) function.

All points belonging to the same swarm have the same identifier. Swarms are then tracked, merged, and eventually ruled-out following the pseudo-code of Figure 10.

In the first section of the algorithm (lines 1–3) we track existing swarms. Every swarm already present in the current image (line 1) is tracked using function OpticalFlow (line 2) derived from [34,35,36,37]. For each swarm, the centroid of all points and the radius of the circle including those points are computed and assigned to fields center and radius of the swarm by function tracking. The average point of each swarm is used to track the swarm itself, using a Kalman filter [42] (line 3).

In the second section of the algorithm (lines 4–10) we generate new swarms. For each cell in the video frame (line 4), if there is a movement (line 5) and there is no swarm in the cell (line 7), the function GoodFeatures [36,37,43,44] is used to create a new swarm (line 8) to be added in the swarm list array. Similarly, if there is a movement (line 5) and the number of points of a swarm within the cell is below a given threshold (line 9), the function GoodFeatures is used to find more points to insert in the point list of existing swarms (line 10).

In the third section of the algorithm (lines 11–17) unstable swarms are excluded from the tracking procedure. In line 11, the algorithm checks whether each tracked swarm has a number of representative points in its support smaller than a threshold (namely threshold${}_{1}$), or the area it covers is larger than another threshold (namely threshold${}_{2}$). In those cases, the swarm is not representative anymore (as it is representing to few points or a too large area) and it is canceled from the set of tracked swarms (line 13). Otherwise, the swarm is compared with all other swarms (line 15) and if its centroid is within the area covered by another swarm (line 16) it is merged (by function merge) with the new swarm. In this case a unique swarm, whose borders include the two swarms original borders, is maintained.

### Tracking Correction

Optical flow tracking, using functions OpticalFlow [34,35], and GoodFeatures [43,44] is prone to errors. A first error situation is represented by points on the scene which do not represent any movements. Those points may be related to abrupt color or shape changes on the background such as manhole covers, railings, etc. Points dynamically tracked may be attracted by those fixed points, and they may become frozen themselves. A second error condition is the one in which different swarms overlap with each other. In this situation, points belonging to a swarm may be attracted by other swarms, changing direction. The effect of such errors is that swarms may become larger and larger because they include points correctly belonging to the tracked object but also points not belonging to it. One possible way to solve this problem is to analyze the swarm centroid at run time, and ruling-out all points whose distances from the centroid are much larger that the distances average value. This solution would be expensive to implement as it should be repeated for all swarms at run-time. To eliminate the first source of problems, our tool creates the so-called hole model before tracking objects, and then it uses this model in all subsequent phases to reduce tracking errors. When building the hole model, the static movement detector (Section 4) works as usual, whereas the dynamic tracking phase (Section 5) manipulates swarms as follows:

- We split the image frame into a cell grid, whose cells are much smaller than the ones introduced in Section 4. We currently use cells of size w=8 and h=8 pixels.
- We record all activities produced by all points of the swarms running on each grid cell. Whenever a swarm size increases beyond a certain threshold, we concentrate on points causing such a size increment. Those points may possibly be anchors within the cell, i.e., formerly moving points, now fixed to the background grid with a high attraction toward other moving points.
- We keep track of all problems and when they appear to be frequent enough on a grid cell, we mark the cell as including anchor points, and, as a consequence, a possible source of tracking errors. On the contrary, cells marked because of transient phenomenon (such as standing people) may be reconsidered as normal cells after some time.

To eliminate all transient phenomenon happening on the video grid, the hole model generation requires several minutes to rule-out all possible inconveniences from the video flow. When the hole model becomes stable, it is not modified any more, and it is used by the tool during the dynamic tracking phase (as indicated by the algorithm of Figure 10) in the following way:

- Every time we create or refresh a new swarm (lines 8 and 10 of Figure 10), we check all points belonging to it. Whenever a point overlap an anchor we rule it out.
- During tracking (line 3 of Figure 10), if a moving point ends-up in an anchor area it is placed in quarantine. Each point in quarantine is checked again for a few subsequent frames. If if is stuck to the anchor, it is ruled-out; otherwise it is maintained. In other words, all points potential causing size increments in a swarm are ruled-out from the swarm before producing any tracking problem.
- Every points not overlapping an anchor is manipulated in the standard way.

## 6. Experimental Results

In this section, we analyze our experimental setting and our experimental results. In more details, Section 6.1 describes our hardware configuration and the benchmarks used for the experiments. Section 6.2 reports considerations on how to trim the tool for tracking accuracy and time efficiency. Section 6.3 discusses our tracking error correction system. Section 6.4 reports data regarding time efficiency. Finally, Section 6.5 presents some comparisons with state of the art tools on publicly available and annotated benchmarks.

### 6.1. Hardware Configuration and Benchmarks

The adopted hardware configuration includes the following devices:

- In each grid node, a video camera Logitech HD Pro Webcam C920. This is a full HD (up to (1920×1080) pixels) camera with H.264 video compression, and automatic low-light correction.
- In all nodes there are CuBox-i4Pro embedded systems [45]. The CuBox-i is a compact micro-computer (a cube of 2” size) with an ARM Cortex quad-core processor, a RAM of 2 GB, a GC2000 GPU, and an external memory (on micro-SD) of 4 GB. In our application it runs the Linux (Ubuntu 16.04-LTS) operating system.
- In the central unit, there is a reference workstation with an Intel i7 860470/2010, 8 MB cache memory, a clock speed of 2.8 G Hz, 4 cores, 8 threads, 8 GBytes of main memory DDR III 1333, and a Windows 7 operating system. We use it to run base-line comparison tests and to collect data coming from different nodes of the embedded intelligence grid.

To reduce all experiments to a common denominator, we implemented a base movement tracking algorithm (BMTA) based on OpenCV corner detection and optical flow analysis. This algorithm manipulates all pixels on each video frame using the goodFeaturesToTrack [36,37,43,44] function plus a few algorithmic optimization presented in [11,30]. We use this tool as a reference point for all comparisons. In this way, we avoid to manually tag and inspect very long video sequences.

**Figure 11:**
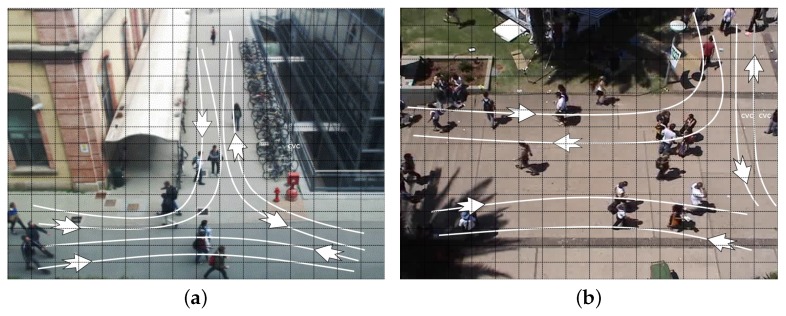
Two typical working scenarios for our application ((**a**) home-made, (**b**) taken from [49]), highlighting six main pedestrian flows which can be used to optimize the divide-and-conquer procedure represented by the grid overlapping both pictures.

**Figure 12:**
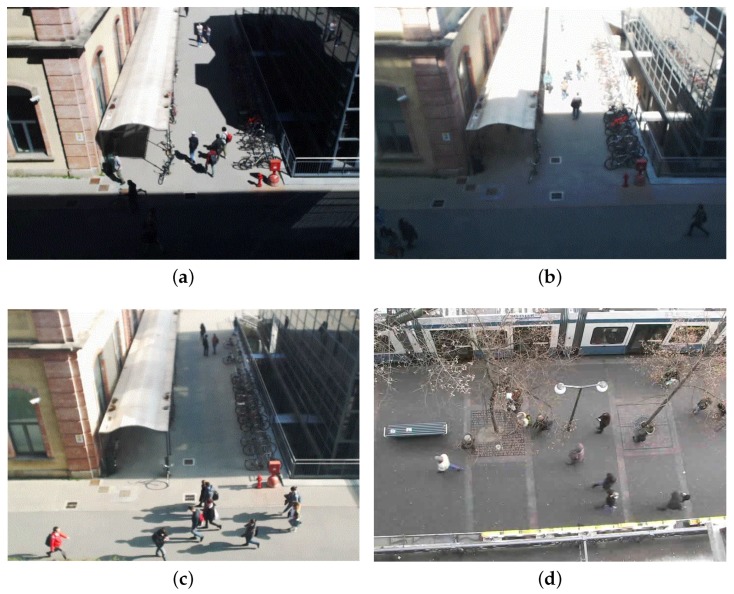
A few working scenarios with different weather conditions and occlusions. (**a**) Very dark building shadows, (**b**) extreme luminosity conditions, (**c**) large and light shadows for all moving objects, and (**d**) tree occlusions.

**Figure 13:**
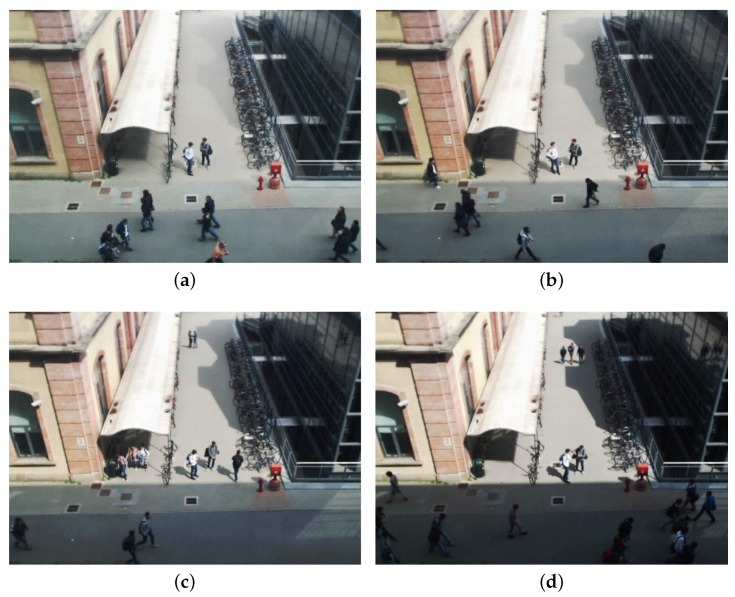
Working conditions with abrupt weather change: From dim (**a**), to sunrise (**b**), bright (**c**). and heat (**d**).

As far as video sequences are concerned, we use home-made generated video sequences to analyze and trim our system, and both home-made and publicly domain benchmarks to evaluate it. Regarding publicly domain benchmarks, we focus on scenarios taken from the BIWI Walking Pedestrians dataset by Pellegrini et al. [46,47], the Crowds-by-Example dataset by Lerner et al. [48,49], and others such as the ones from Roth et al. [50] or Riemenschneider [51]. Even if those videos are based on different standards and formats, we transformed and manipulated all of them as VGA videos with 640×480 pixels. Figure 11 shows two typical working scenarios for our application in which a camera, placed at the second floor of a building, it is framing a pedestrian intersection area. The one on the left-hand side is home-made, and it is a quite heterogeneous, as it includes two buildings and a long white canopy. Notice that the two buildings have different colors, and the one on the right-hand side of the picture is covered by reflecting dark glasses. The one on the right-hand side is taken from [49] (the University Students dataset), and it includes very crowded areas. Both pictures highlight all main (six overall) pedestrian flows, partially overlapping and hidden by other artifacts, and the grid generated by our divide-and-conquer strategy and used in all application phases. Those flows can be used by function select (see the beginning of Section 4) to optimize our divide-and-conquer strategy. The same area of Figure 11a appears in Figure 12 with very dark building shadows (a), extreme luminosity conditions (b), and large and light shadows for all moving objects (c). Figure 12d (taken from the seq_hotel data set of [47]) shows moving trees and branches which partially occlude the analyzed area. Again in the same scenario, Figure 13 shows a situation in which the weather is changing very rapidly (notice the same party of two people talking at the corner of the white canopy) from a slightly somber overcast situation to a highly bright and shaded condition. The system has to adapt to this change to balance the rumors, that shadows and bright lights, add to the scene.

### 6.2. Parameters Setting

In this subsection, we mainly analyze the effects of our divide-and-conquer strategy on a video sequence including 15,000 frames (about 10 min).

Table 1 shows how we select the value of *N* and *M* to partition the image frame *F* into the partitioned frame $F_{P}$ (see Figure 1). The table reports some statistics as a function of *N* and *M*, using in all cases the maximum possible refreshing rate (i.e., SIZE=N×M). Column Cells reports the number of cells (out of N×M) on which the tool detects movement, column # Pixels the corresponding area in terms of pixels, and column % Pixels the ratio between this last number and the total number of pixels in the image. Finally, column % Identification Error reports the error of the static tracking phase, i.e., the percentage number of frames in which the tool fails to report a movement detected by the BMTA algorithm. Notice that those errors are further reduced by the dynamic tracking and error correction system. If we analyze column % Pixels, we realize that from N=M=10 to N=M=20 the algorithm has a quite steady behavior and it detects all moving pixels (about 50% overall) within the working area. For larger cells, it overestimates the number of pixels belonging to cells on which a movement has been detected. For smaller cells, it underestimates it, because the cells include so little information that module DLD produces useless histograms. At the same time, column % Identification Error shows how movement detection errors tend to reach a minimum around N=M=16. Those considerations motivate our decision to select N=M=16 for all subsequent experiments.

**Figure 14:**
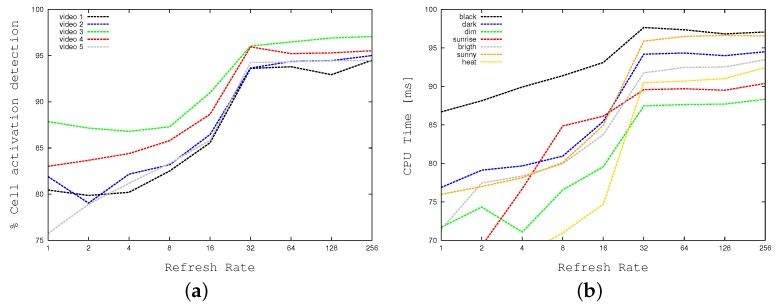
Cell activation detection as a function of the refresh rate: (**a**) Standard weather, (**b**) Black, dark, dim, sunrise, bright, sunny, heat weather conditions.

Table 2 shows the impact of the refreshing rate (i.e., SIZE) in our computations. As previously described, columns SIZE and Refreshing Rate indicate the refreshing rate (i.e., with SIZE=1 every cell is manipulated every 256 frames, whereas with SIZE=256 every cell is manipulated at every frame). Column % Cell activation detection shows the ratio between the movement as detected using our cell strategy and the one detected by the reference algorithm BMTA. For example, with the maximum refreshing rate of SIZE=256, the tools detects 94.9% of BMTA movements (i.e., 100%−5.1%, which is the error reported by Table 1 for N=M=16), whereas with SIZE=16 it detects only the 87.5%. This column shows that the tool is able to catch almost all grid movements with a SIZE larger than 16. At the same time columns CPU Time and FPS show how CPU times (in milliseconds) and frame-per-seconds vary as a function of the refreshing rate. To have a more intuitive and graphical representation of the relationship among the cell activation and the refreshing rate, Figure 14 plots the first a function of the last one. Figure 14a concentrates on standard weather conditions, whereas Figure 14b focuses on more adverse conditions. Table 2 and Figure 14 show that the best compromise is reached for a refreshing rate equal to 8 frames (SIZE=32), which is also the value we use in our standard setting.

### 6.3. Dynamic Tracking Error Correction

**Figure 15:**
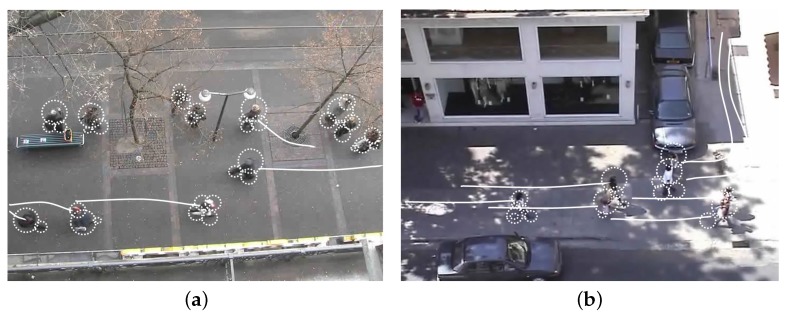
Relationship between computed swarms and movement annotations, for two videos taken from [47] (**a**), and [49] (**b**). Our swarms proved to cover annotations in a complete and constant way along the video.

Table 3 shows data coming from our dynamic tracking system (Section 5), showing the impact on result accuracy of our hole model and tracking correction system. We present some results on the same video used in Section 6.2. As previously described, column SIZE indicates the complement of the refreshing rate. Columns # Pixels reports the average number of support points of each swarm (column Support), and the number of BMTA pixels covered by the same swarm (column Covered). This implies that with a very small number of points (about three) each swarm is able to represent movements described by a large number of BMTA points (up to 50). Column # Swarms per Point indicates the number of swarms which cover each single BMTA point on each video frame. This column indicates that from SIZE>8 each point is covered by at least one swarm for its entire life. For the sake of exemplification, Figure 15 illustrates movement annotations (white lines), as given by the original authors, and the swarms generated by the system (white dotted circles) on two different video frames taken from [47,49]. It is possible to notice that many swarms cover each annotation, whereas other are really close to them.

The next four columns compare data with and without the hole model. Columns 5 and 6 report the number of swarms which have been active for at least a minimum number of video frames (about 10), with and without our hole model, respectively. As indicated by the pseudo-code of Figure 10 (lines 12–13) when a swarm deteriorates (i.e., its number of support points goes below a certain threshold or its size increases too much) it is canceled from the swarm list. As columns 6 reports a much higher number of swarms compared to column 5, the hole model proved to be quite effective in stabilizing swarms and point tracking. A similar consideration holds analyzing columns 7 and 8, reporting (in percentage) the area overlap among different swarms. Again without the hole model, swarms do overlap much more indicating a higher level of instability and redundancy, coupled with a smaller representativity and duration (columns 5 and 6).

**Figure 16:**
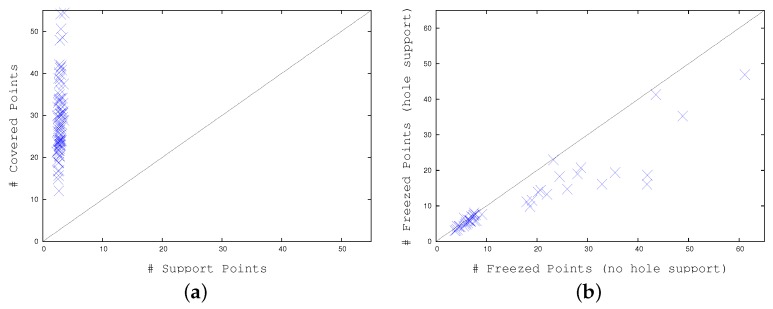
(**a**) Number of support points versus number of base movement tracking algorithm (BMTA)-represented points by a single swarm. (**b**) Number of frozen points with no hole support versus number of frozen points with hole support.

Following Table 3, and considering several video frames, Figure 16a plots column 3 with respect to column 2 of Table 3, i.e., the number of covered points for each swarm versus the number of support points. Again, as the first value is about 10 times larger than the second, we are able to represent with a low effort a large number of moving points. Similarly, Figure 16b plots the number of swarm points that remain “frozen”, i.e., stuck on some image key-point, with and without our error correction. In this case, the first set of values is about 30% smaller than the second one, showing the same error correction ability.

### 6.4. Time Efficiency

In this sub-section we present the running times of our application using different settings.

**Figure 17:**
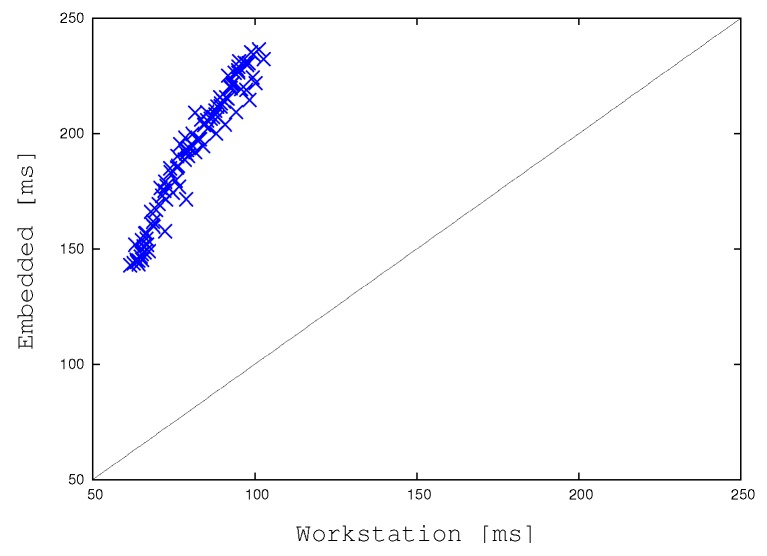
Reference algorithm: Comparing running times on the desktop workstation against the ones gathered on the embedded system.

Figure 17 compares the “wall-clock times” of the reference strategy (BMTA) while running on our desktop workstation with the one adapted to run on our embedded system. Notice that the wall-clock time is the time necessary to a (mono-thread or multi-thread) process to complete its job on a new input image, i.e., the difference between the time at which an image is completely handled and the time at which this task started. For this reason, the wall-clock time is also known as “elapsed time”. The plot shows that the embedded system is about three times slower than the workstation to run the same reference algorithm, and it is able to deal with a single video frame in a time ranging from about 140 to about 240 milliseconds ([ms]). The slowest time entails about 5 frames per seconds which, as stated right in the introduction, are not adequate for a real-time application and motivate our work. The workstation requires from about 60 to 100 milliseconds per frame, allowing at least 10 frames per second.

**Figure 18:**
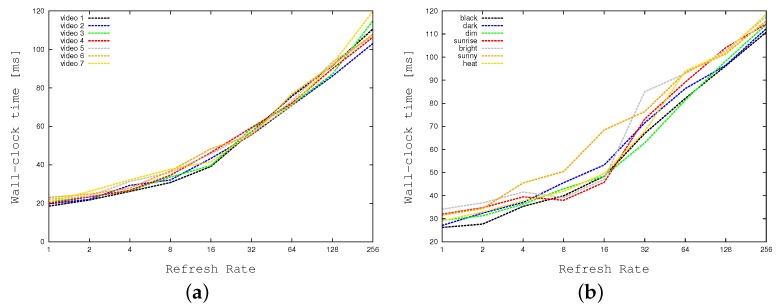
Computation times of our implementation (with no multi-threading) as a function of the refresh rate on the embedded system. Standard (**a**), and extreme (black, dark, dim, sunrise, bright, sunny, heat) weather conditions (**b**).

**Figure 19:**
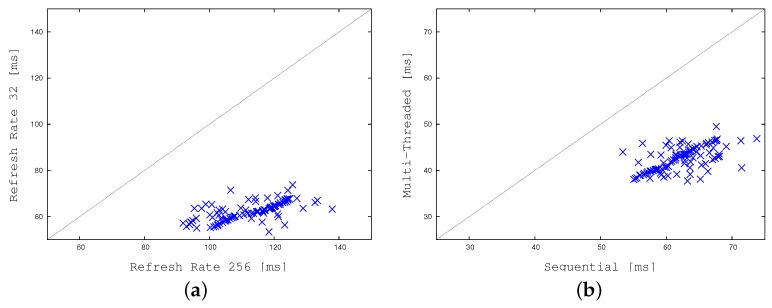
Comparing computation times on the embedded system: (**a**) The sequential implementation with two different refresh rates, and (**b**) The multi-threaded version versus the sequential one.

Figure 18 and Figure 19 demonstrate the impact of our divide-and-conquer strategy in terms of time efficiency. Figure 18 plots the wall-clock times required by our application when running on the embedded system in sequential mode (i.e., with multi-threading disabled) as a function of the refreshing rate. As before, all CPU times are expressed in milliseconds. Figure 18a concentrates on standard weather conditions, whereas Figure 18b focuses on more adverse weather conditions, and crowded contexts, requiring larger times. The two graphs prove that the divide-and-conquer strategy may reduce elapsed times by a factor of 2. As previously described, to obtain the right balance between time efficiency and detection accuracy, we selected a refresh rate equal to 32 cells per frame. Figure 19a compares our tool using this refreshing rate with the highest possible one. The graph shows a gain of a factor of about 2. Figure 19b relates wall clock-times of the multi-threaded implementation with the ones of the sequential version, both of them running with a refresh rate of 32. The concurrent version (running 5 threads) is about two times faster on average.

**Figure 20:**
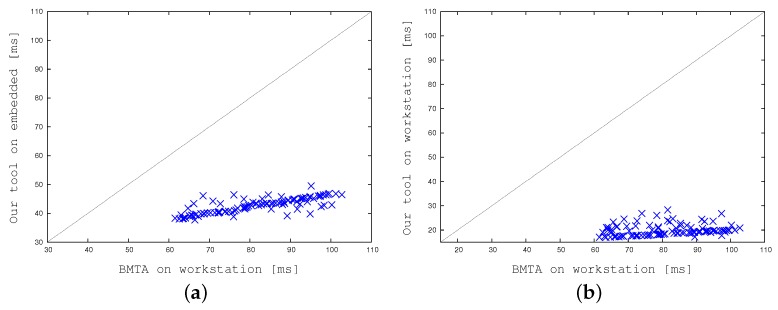
Comparing computation times: BMTA running on a desktop workstation versus our divide-and-conquer concurrent approach running on the embedded system (**a**), and the desktop workstation (**b**).

Finally, Figure 20 compares BMTA with our multi-threaded strategy with the selected cell size and refreshing rate. In Figure 20a the reference version runs on the desktop workstation whereas our implementation runs on the embedded system. Our implementation proved to be about 1.9 times faster. In Figure 20b both the reference and our tool run on the desktop workstation. In this case, representing the final apple-to-apple comparison, our implementation is about 4–5 times faster. This definitely enables run-times embedded applications.

### 6.5. Comparison with the State-of-the-Art Tools on Standard Benchmarks

In this section we compare our results, on publicly available benchmarks, with the performance of some prior state-of-the-art techniques.

We use the following data-sets: BIWI Walking Pedestrians data-set by Pellegrini et al. [46,47] (seq_eth and seq_hotel), and Crowds-by-Example by Lerner et al. [48,49] (Zara01, Zara02, and University Students). All these sequences are captured from a more or less aerial and oblique views. Those benchmarks have been used by many authors [52,53,54,55,56,57,58] for different purposes from tracking to human behavior prediction in crowded scenes.

We compare the accuracy (in terms of pedestrians tracked in the video sequence) and speed (in terms of frames per second) of the following online algorithms: Online Boosting [59], KMS [60], SMS [61], ASLA [62], Frag [63]. and AdaPT [54]. Table 4 reports our data. The first row indicates the total number of pedestrians in the corresponding video sequence. All other rows report the accuracy, and the maximum number of frame per seconds, for all strategies. For our method, we show both the workstation and the embedded system frame rates (the accuracy values are the same).

In summary, we can make the two following observations. First of all, it can be noticed that our implementation obtains comparable (if not better) results in terms of accuracy. This is somehow unexpected, and it may be due to the fact that all other methods are all not properly trimmed for the analyzed scenarios. This consideration can be partially supported by the relative low detection rate, even if this can be balanced by the complexity of those scenarios. Secondly, our tools proved to be much faster then all other techniques, especially when comparing the version running on the workstation. This is somehow not surprising, time efficiency being one of the main targets of our work. Moreover, the advantage of the workstation with respect to the embedded system is somehow not as high as expected, and this is partially due to the fact that the application was born, and it has been explicitly optimized, for the embedded system.

## 7. Conclusions

This work presents a system for moving object exposure, focusing on pedestrian detection, in external, unfriendly and heterogeneous environments. The system orchestrates well-known robust and efficient detection and tracking techniques to obtain good results in diversified scenarios, and its power relies more on the overall receipt than on specific algorithmic details. The application is designed to work with fixed and inexpensive off-the-shelf video cameras mounted at a few meters from the ground, and to run on embedded systems with finite computational resources. Experimental results on several video sequences show robustness and accuracy of the overall detection strategy beyond current state-of-the-art approaches, and enables real-time applications on inexpensive embedded units of those technologies.

## Figures and Tables

**Table 1:** Number of moving pixels (cells) detected as a function of the partitioning thresholds *N* and *M*.

N	M	# Cells	# Pixels	% Pixels	% Identification Error
4	4	10	192,000	62.5	22.4
8	4	19	182,400	59.3	18.8
8	8	37	177,600	57.9	14.3
10	8	45	172,800	56.2	13.1
10	10	58	165,888	54.0	10.2
14	10	78	157,248	51.1	6.8
16	16	128	153,600	50.0	5.1
20	16	162	155,520	50.6	6.2
20	20	209	160,512	52.2	8.4
28	20	272	143,616	46.7	11.0
32	32	459	137,700	44.8	19.4
40	40	687	131,904	42.9	22.8
64	48	1250	125,000	40.6	36.1

**Table 2:** Cell activation detection error as a function of the refresh rate.

SIZE	Refreshing Rate	% Cell Activation Detection	CPU Time [ms]	FPS
1	256	78.5	46.15	22
2	128	81.7	47.19	21
4	64	82.9	49.25	20
8	32	84.4	55.93	18
16	16	87.5	57.51	17
32	8	93.9	58.11	17
64	4	94.2	73.31	13
128	2	94.6	88.97	11
256	1	94.9	105.33	9

**Table 3:** Swarm statistics.

SIZE	# Pixels		# Swarms per Point	# Swarms		Swarm Overlap	
	Support	Covered	[%]	No Hole Model	Hole Model	No Hole Model	Hole Model
1	3.05	50.59	0.25	143	178	0.30	0.17
2	3.61	54.38	0.66	199	298	0.60	0.47
4	3.34	48.53	0.80	291	470	0.79	0.54
8	3.10	41.81	0.90	421	699	1.03	0.61
16	2.91	41.24	1.10	501	950	1.14	0.75
32	3.00	39.71	1.35	540	1143	1.48	0.97
64	2.86	38.69	1.37	648	1357	1.67	1.08
128	2.75	33.60	1.33	758	1557	1.91	1.12
256	2.68	33.45	1.44	817	1711	1.97	1.20

**Table 4:** Accuracy (in terms of pedestrians tracked in the video sequence) and speed (in terms of frames per second) of the following online algorithms: Our Method compared to Online Boosting [59], KMS [60], SMS [61], ASLA [62], Frag [63], and AdaPT [54].

	seq_eth		seq_hotel		Zara01		Zara02		University Students	
	Acc	FPS	Acc	FPS	Acc	FPS	Acc	FPS	Acc	FPS
Total Number of Pedestrians	367		420		148		204		434	
Our Method (on Workstation)	258	59	295	57	104	58	143	58	303	53
Our Method (on Embedded)		27		26		26		27		21
Online Boosting	241	26	258	27	61	27	65	27	278	25
KMS	259	7	287	7	69	7	72	7	291	7
SMS	98	33	112	35	32	35	33	35	226	32
ASLA	201	16	220	16	54	16	57	17	294	15
Frag	262	8	265	8	68	7	70	7	224	7
AdaPT	178	20	156	19	36	21	32	19	281	18
